# Five Forms of Coerced “Self-Produced” Child Sexual Exploitation Material: A Critical Interpretive Synthesis

**DOI:** 10.1177/15248380241271376

**Published:** 2024-09-08

**Authors:** Genevieve Bloxsom, Gemma McKibbin, Cathy Humphreys, Jennifer Davidson, Nick Halfpenny

**Affiliations:** 1 University of Melbourne, VIC, Australia; 2 MacKillop Family Services, Melbourne, VIC, Australia

**Keywords:** sexual abuse, child abuse, child abusers, prevention of child abuse

## Abstract

This review explored how the phenomenon of coerced “self-produced” child sexual exploitation material (CSEM) has been constructed in the literature using Critical Interpretative Synthesis. Selected keywords were systematically searched on relevant databases. Types of papers included were: peer-reviewed research articles; conceptual papers; commentary papers; theses; book chapters; systematic reviews; and government reports. Papers published in English between January 2005 and November 2022 were included. The initial search revealed 1,021 papers, after two reviewers applied the inclusion and exclusion criteria, 38 papers were selected for the final sample. Findings indicated five forms of coerced “self-produced” CSEM: Solicitation; Peer Sexting; Viral Challenge; Sextortion; and Financial Coercion. The forms are described and critically analyzed through an “Accountability Lens.” This Lens was developed to be victim-centered including identifying the coercive actions of the person responsible for the exploitation. The review found an absence of a consistent victim-centered approach to how the phenomena of coerced “self-produced” CSEM is understood that would ensure children are not held responsible for being exploited.

## Introduction

Child sexual exploitation involves a perpetrator making a child believe they will be given something of value in return for being a victim of sexual abuse. This could include gifts, money, accommodation, food, alcohol, drugs, a “relationship,” “love,” improved social status, increased social media responses, or any other type of benefit (The Children’s Society, 2023). The person responsible for the exploitation may also threaten the child, and then offer short-term safety or conditional privacy in return for being a victim of sexual abuse (Wolak et al., 2018). This review focuses on “self-produced” child sexual exploitation material (CSEM), which refers to sexual photos, videos, or live streaming of children that have been created by the child.

Mathews and Collin-Vézina’s (2019) conceptual model for defining child sexual abuse demonstrates how the emerging phenomenon of “self-produced” CSEM is a form of child sexual abuse. The model stipulates four separate conditions that need to be met for child sexual abuse to occur: (a) That the person is either developmentally a child or legally a child; (b) That the child’s true consent is absent, due to being developmentally unable, legally unable or the child did not give consent; (c) That the act is sexual and either the abuser or another person receives sexual or mental gratification, or the act is legitimately experienced by the child as a sexual act; and (d) The act constitutes abuse by occurring within a relationship of power, while the child is in a position of inequality or through exploiting the child’s vulnerability. The phenomenon of children being coerced to “self-produced” CSEM and provide this to the person responsible for the exploitation meets these four conditions.

### The Phenomena Emerging Over Time

Children coerced to “self-produce” CSEM appears to be increasingly common. In 2000, Finkelhor et al. reported the results of a youth internet safety survey in which the definitions of online victimization only included requests by perpetrators for a child to engage in sexual talk or meet in person. There was no consideration of the possibility children could be coerced to “self-produce” CSEM. Despite the phenomena not being detected in academic literature at this time, media reports in 2005 confirmed a case of coerced “self-produced” CSEM dating back to 2000 (Eichenwald, 2005; Winfrey & Terry, 2006).

Within 2 years, understanding in this area moved from the construction of an “isolated incident” to more widespread phenomena of concern. A survey by Mitchell et al. (2007) involving 1,500 children in the United States found that 4% of participants aged 12 to 17 years old had been asked on the internet to “self-produce” CSEM. A year later, an online study (Power to Decide, 2008) involving 653 people aged 13 to 19 found that 20% had posted or shared naked or semi-naked images or videos of themselves.

The Internet Watch Foundation identified the phenomena of “self-generated” CSEM for the first time in their 2012 annual report, which indicated 12,224 “self-produced” images of children had been reported (Internet Watch Foundation, 2012). Three years after this report, Crofts et al. (2015) conducted an online survey (*n* = 1,416) and focus groups with children in Australia and found that 38% of participants aged 13 to 15 and 50% of participants aged 16 to 18 had sent a sexual picture or video to somebody else. The 2018 Netclean Report (Baines, 2019) reported a survey of 272 police officers across 30 countries and found over 90% said it was common or very common for investigations to involve “voluntary self-produced” CSEM, and over 75% said it was common or very common for investigations to involve child sexual exploitation images that are the result of grooming and sextortion. This same year, Gewirtz-Meydan et al.’s (2018) study revealed the majority of the 1,560 children who were surveyed believed it was extremely likely sexting would create trouble at school (71%), result in trouble with the police (59%), negatively impact chances of future employment (61%) and hurt friendships (57%) and family (66%).

In 2019, the Internet Watch Foundation reported 38,424 web pages containing “self-produced” CSEM (Internet Watch Foundation, 2019). During the Coronavirus pandemic this figure increased by 374% within 2 years and in 2021 the Internet Watch Foundation identified 182,281 web pages containing images or videos of “self-produced” CSEM. The following year Ringrose et al. (2022) found that 74% of teenage girls (*n* = 100) who participated in focus groups had previously been asked to provide a nude image to somebody. The annual report of the Internet Watch Foundation (2022) recorded a 9% annual increase in webpages containing “self-produced” CSEM, totaling 199,363 and representing 78% of child sexual exploitation webpages reported in 2022. “Self-produced” CSEM has become the most common type of child abuse material now to be reported (Internet Watch Foundation, 2022).

These statistics reveal that children being coerced to “self-produce” CSEM is rapidly increasing and requires further exploration. A study by Finkelhor et al. (2023) analyzed 245 questionnaire responses that disclosed childhood episodes of “self-produced” CSEM and found the way “self-produced” CSEM was shared influenced the negative impact children experienced. This review continues the inquiry of “self-produced” CSEM and how it is constructed in the literature. Specifically, the review identified different ways children are coerced, highlighting the transition from victim-blaming to victim-centered constructions.

### Language Considerations

In the evolving area of child sexual exploitation, many terms are yet to achieve agreement among international experts (Terminology and Semantics Interagency Working Group on Sexual Exploitation of Children, 2016). Throughout the papers considered in this review, multiple different terms have been used to describe the same concepts. In this paper, we use the term “child sexual exploitation material” (CSEM) and view this as a broad term to describe child sexual exploitation imagery, videos or live streaming. The term “child pornography” appears in literature and the phrase “children in pornographic material” is used in some legislation (United Nations, 1989). We do not adopt these terms because we consider that describing child exploitation as pornography normalizes and minimizes the severity of sexual abuse (Clough, 2012). The CSEM explored in this review focuses on children who have “self-produced” this material. We consider that the term “self-produce,” or alternatives such as “self-generate” are misleading, and can imply that children are responsible for the abuse (Internet Watch Foundation, 2012). Nevertheless, we use the term “self-produced” in the absence of a more suitable term. In an attempt to minimize the risk of the term “self-produced” being victim-blaming, a description of perpetrators’ coercive behavior is included where possible to ensure their actions remain visible.

The term “perpetrator” is used throughout this review to describe adults who have coerced children to “self-produce” CSEM. Some papers in this review use the term “perpetrator” to describe children, but we assert that viewing children as “perpetrators” fails to recognize their capacity for behavioral change and their developmental difference from adults (Ghani, 2016). To ensure this review remains child-focused, we do not use the term “perpetrator” to describe children. In instances where children have leveraged a power imbalance to encourage another child to “self-produce” CSEM, we use the term “harmful sexual behaviour” (Hackett et al., 2019). For clarity, we understand children as individuals under the age of 18.

### Legal Responses

Globally, there is a lack of consistent legal responses to “self-produced” CSEM. Many countries, including Australia, New Zealand, South Africa, the United States, and the United Kingdom, vary when deciding if a child should be prosecuted for “self-producing” CSEM and what legal defenses should be available (O’Connor et al., 2017). For example, legislation in New South Wales (Australia) provides a defense to producing child abuse material if the only person depicted in the material is the accused person (Crimes Act 1900 (NSW) s. 91HA); this is contradicted by Australian Commonwealth Law which enables a child who takes sexual photos of themselves to be guilty of a child sex offense (Criminal Code Act 1995 (Cth) s. 474.19(1)). In the United Kingdom, it is a crime for anybody under the age of 18 to take a sexual photo of themselves (Avon & Somerset Police, 2014). In the United States, each state has a different response to sexting and the penalty can range from a petty offense, misdemeanor or felony, with many states punishing the child by including them on the sex offender registry (O’Connor et al., 2017). While there is debate regarding the legal, moral, and ethical implications of the child’s actions when they voluntarily or by coercion “self-produced” CSEM, this review focuses solely on coerced “self-produced” CSEM where the children are victims. The authors’ stance is that children who are victims of coerced “self-produced” CSEM should receive support, not punishment through criminalization.

## Methodology

The literature focusing on the phenomenon of coerced “self-produced” CSEM contains complex and diverse points of view. To understand the existing literature, a methodology capable of examining this challenging and emotionally laden topic was employed—Critical Interpretive Synthesis (CIS) developed by Dixon-Woods et al. (2006). The stages of the CIS involved: an iterative construction of the research question; a search and selection of relevant literature; the extraction of data relevant to the phenomena; several stages of coding to analyze the extracted data; and finally consultation with subject matter experts to critique and synthesize the data. Theories relating to the phenomena evolved alongside all stages of the CIS. The research question informing the review was: *How is the phenomenon of coerced “self-produced” CSEM by children constructed in the literature?*

### Paper Selection and Inclusion

A systematic search of the evidence was undertaken in November 2022. The search strategy identified relevant primary search term groups and alternative search terms. The following electronic databases were searched: ASSIA, CINAHL, Cochrane Database of Systematic Reviews, ERIC, Family & Society Studies Worldwide, Medline, and PsychINFO. Google Scholar was also searched and the first 200 papers in the results were reviewed, as recommended by Haddaway et al. (2015), relevant publications were selected based on the inclusion and exclusion criteria. Table 1 provides the search terms used for the CINAHL database. Slight variations were made for other databases as the truncation settings varied.

**Table 1.** Search Terms Used for CINAHL Database.

Primary search term groups	Search terms	Paper location
A person under the age of 18	Child* OR (young people) OR Teen* OR Adolescent* OR Kid* OR Underage OR Minor*	Only abstract and title
Actions the child was made to do	self-produce* OR self-generate* OR youth-produce* OR Creat* OR Post* OR camming	Only abstract and title
Perpetrator’s actions	Sext* OR Coerce* OR Manipulate* OR Groom* OR Sexual trade OR Selfexploitation OR Child sexual abuse OR sextortion OR Trafficking	Full text
The child exploitation content that is created as a result	Child sexual abuse material* OR image-based adj2 abuse* OR Sexual* explicit content OR Child* porn* OR live online child* sex* abuse OR Child* exploitat* material OR CSEM OR CSEM	Full text

The inclusion criteria focused on literature relating to children who have been coerced to “self-produce” CSEM, whereby the person responsible communicated directly with the child. Table 2 provides the inclusion and exclusion criteria that were used for the final search. The search included papers published in English between January 2005 and November 2022. The rationale for searching papers from January 2005 related to the technological advancements and media stories occurring at that time, which included the launch of video-sharing website YouTube (Mayor, 2022) and the rapid increase in the use of the social media site Myspace (Jackson & Madrigal, 2011). The authors’ believe that before 2005, the phenomena of coerced “self-produced” CSEM was not known to the general public, and therefore there would not be relevant papers published that met the inclusion criteria for this review.

**Table 2.** Inclusion and Exclusion Criteria Used in Screening and Selection of Papers.

Inclusion	Exclusion
Addresses online child exploitation of people under the age of 18	Did not address online sexual exploitation of people under the age of 18.
Addresses children who have been coerced to “self-produce” CSEM whereby the offender communicated directly with the child and there was no facilitator organizing the abuse and/or receiving payment. In other words, a child is providing imagery directly to a person, where the social or economic capital goes directly to the child.	Publications that only addressed CSEM that were developed without the child’s knowledge (for example digitally manipulating photos of children to create child exploitation material, without the child’s knowledge or filming children without the child knowing).
Addresses children (age under 18) sexting, that included an element of coercion, pressure or manipulation.	Only discussed purely consensual sexting between young people.
Both peer review literature and gray literature will be included.	Book reviews, magazine articles, news articles, social media posts, podcast transcripts, poems and posters.
Publications between January 1, 2005 and November 28, 2022.	

*Note*. CSEM = child sexual exploitation material.

**Figure 1.**
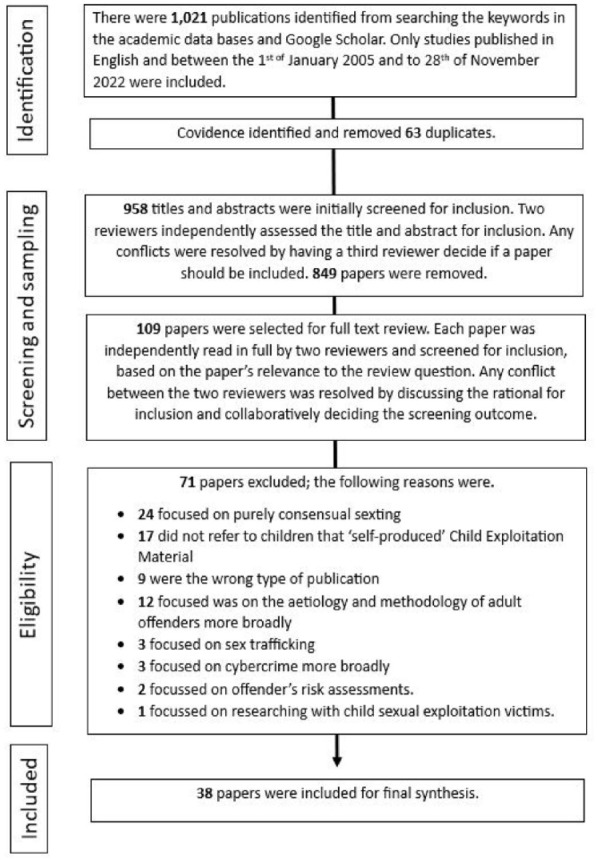
Paper selection process.

When the search was complete, papers were exported into Covidence to streamline the screening process. Covidence software automatically identified and removed duplicate papers supported two reviewers to independently screen the title and abstracts and kept a record of the decision-making process (Babineau, 2014). When conflicts arose, a third reviewer decided if the paper would progress to full-text review. The papers included in the full-text review were independently read by two reviewers and assessed in relation to the inclusion criteria. All conflicts that occurred were resolved between the two reviewers through consultations and collaborative decision making with the third reviewer. This process is outlined in Figure 1.

Thirty-eight papers were included in the final synthesis (see Table 3). These papers included quantitative and qualitative research articles, conceptual papers, commentary papers, dissertations, theses, book chapters, systematic reviews and government reports. The papers were read in full, and the relevant information was extracted and then exported into NVivo (Release 1.7.1). NVivo was used to create and organize digital codes within the extracted information, as part of the analysis and synthesis process.

**Table 3.** Papers Included in Final Synthesis (38).

Author, year	Paper title	Country	Study design	Sample (*N*)	Age (years)
Açar (2017)	Webcam child prostitution: An exploration of current and futuristic methods of detection	Türkiye	Opinion article	Not applicable	Not applicable
Barr (2016)	Child autopornography in the age of Snapchat-an affirmative defense	United States	Commentary article	Not applicable	Not applicable
Caffo et al. (2021)	Child abuse and exploitation: What we know about the problem and new perspectives	Italy	Book chapter	Not applicable	Not defined
Cooper et al. (2016)	Adolescents and self-taken sexual images	Scotland	Systematic literature review	88 studies	Not applicable
Mitchell et al. (2007)	Online requests for sexual pictures from youth: Risk factors and incident characteristics	United States	Qualitative study: Nationally telephone survey	1,500 participants	10–17
Finkelhor et al. (2022)	Prevalence of online sexual offenses against children in the US	United States	Quantitative study: Online survey	2,639 participants	18–28
Gámez-Guadix et al. (2022)	Assessing image-based sexual abuse: Measurement, prevalence, and temporal stability of sextortion and nonconsensual sexting (“revenge porn”) among adolescents	Spain	Quantitative study: Self-report instruments	1,820 participants	12–17
Giroux (2021)	The phenomenon of self-generated sexual content among adolescents	Netherlands	Qualitative study. Desk review of literature	Not applicable	Not applicable
Grannò et al. (2020)	Denouncing child manipulation to produce sexualized imagery on YouTube	United Kingdom	Mixed methods study: Semantic analysis	1,398 YouTube comments.	Not applicable
Graw Leary (2008)	Self-produced child pornography: The appropriate societal response to juvenile self-sexual exploitation response to juvenile self-sexual exploitation	United States	Scholarly article: Review of the significant doctrinal basis for governmental intervention	Not applicable	Not applicable
Hasinoff (2010)	No right to sext? A critical examination of media and legal debates about teenage girls’ sexual agency in the digital age	United States	Qualitative study: Analyzed a range of texts	Not defined	Not applicable
Jonsson et al. (2015)	Online sexual behaviors among Swedish youth: Associations to background factors, behaviors and abuse	Sweden	Quantitative study: Questionnaire	3,503 participants	High school students with undefined age
Judge and Saleh (2013)	Sexting, cybersex, and internet use: The relationship between adolescent sexual behavior and electronic technologies	United States	Book chapter	Not applicable	Not applicable
Karaian (2012)	Lolita speaks: “Sexting,” teenage girls and the law	Canada	Qualitative study: Post-structural analysis of previous studies	2 online studies	Not applicable
Levine (2022)	Increasing the efficacy of investigations of online child sexual exploitation: Report to Congress	United States	Mixed methods study: Analysis of online child exploitation cases	Not applicable	Not applicable
Lievens (2012)	Risks for young users on social network sites and the legal framework: Match or mismatch?	Austria	Qualitative study: Evaluates the current legal framework for (cyber)bullying and sexting	Not applicable	Not applicable
Macilotti (2020)	Online child pornography: Conceptual issues and law enforcement challenges	France	Book chapter	Not applicable	Not applicable
Meehan (2022)	“If someone’s freaky, everyone loves it. It’s all about the drama”: Young women’s responses and reactions to image-based sexual abuse of other young women	New Zealand	Qualitative study: Focus groups	106	12–16
Moritz and Christensen (2020)	When sexting conflicts with child sexual abuse material: the legal and social consequences for children	Australia	Journal article: Explores socio-legal considerations arising when older children possess and share intimate online material	Not applicable	Not applicable
O’Malley and Holt (2022)	Cyber sextortion: An exploratory analysis of different perpetrators engaging in a similar crime	United States	Qualitative study: Content analysis	152 offender files	18+
Patel and Roesch (2022)	The prevalence of technology-facilitated sexual violence: A meta-analysis and systematic review	Canada	A meta-analysis and systematic review	25 papers	Not applicable
Prat and Jonas (2013)	Psychopathological characteristics of child pornographers and their victims: A literature review (English and French)	France	Systematic literature review	34 papers	Not applicable
Primack (2018)	Youth sexting and the first amendment: Rhetoric and child pornography doctrine in the age of translation	United States	Qualitative study: Rhetorical translation to review Supreme Court doctrine	4 Supreme Court cases	Not defined
Quayle (2021)	Affordances, social media and the criminogenic nature of the Internet: Technology-mediated child sexual abuse	Scotland	Book chapter	Not applicable	Not defined
Ringrose et al. (2022)	Young people’s experiences of image-based sexual harassment and abuse in England and Canada: Toward a feminist framing of technologically facilitated sexual violence	United Kingdom	Qualitative study: Focus groups	206	11–19
Ringrose et al. (2021)	“Wanna trade?”: Cisheteronormative homosocial masculinity and the normalization of abuse in youth digital sexual image exchange	United Kingdom	Qualitative study: Focus groups	144	11–18
Salter and Hanson (2021)	“I need you all to understand how pervasive this issue is”: User efforts to regulate child sexual offending on social media	Australia	Book chapter	Not applicable	Not applicable
Seto (2013)	Solicitation	Canada	Book chapter	Not applicable	Not applicable
Slane (2013)	Sexting and the law in Canada	Canada	Commentary article	Not applicable	Not applicable
Smallbone and Wortley (2017)	Preventing child sexual abuse online	United Kingdom	Book chapter	Not applicable	Not applicable
Strasburger et al. (2019)	Teenagers, sexting, and the law	United States	Opinion article	Not applicable	Not applicable
Thomas (2018)	“What should I do?”: Young women’s reported dilemmas with nude photographs	United States	Qualitative study: Inductive thematic analysis	462 stories posted on a website	Not defined
Thorburn et al. (2021)	To send or not to send nudes: New Zealand girls critically discuss the contradictory gendered pressures of teenage sexting	New Zealand	Qualitative study: Workshops	28 participants	16–17
United States Department of Justice (2016)	The national strategy for child exploitation prevention and interdiction	United States	A report to congress: A year of inter-agency working-group discussion	Not applicable	Not applicable
Sheehan (2016)	Producers of indecent images of children: A qualitative analysis of the etiology and development of their offending patterns	United Kingdom	Qualitative study: Interviews and case study analysis	22	18+
Villacampa (2017)	Teen sexting: Prevalence, characteristics and legal treatment	Spain	Mixed methods: Questionnaire	489	14–18
Whittle and Hamilton-Giachritsis (2017)	Offender behavior	United Kingdom	Book chapter	Not defined	Not defined
Wolak et al. (2012)	How often are teens arrested for sexting? Data from a national sample of police cases	United States	Quantitative study: Mail surveys and telephone interviews	Surveys (2,712), Interviews (675)	18+

### Analysis

Thematic analysis was undertaken using the method set out by Braun and Clarke (2006). The extracted data was read several times by the first author to establish familiarity with the content. Initial coding involved the first author attaching short phrases to pieces of text, generating over 600 codes. The first author grouped initial codes into focus codes, which formed emerging thematic categories. Together, all the authors reviewed the thematic categories to formulate an answer to the review question. Five different constructions of how children are coerced to “self-produced” CSEM became evident.

A critical lens was then applied to the data. We could not identify an existing critical lens well-suited to the topic so the authors developed the “Accountability Lens.” This lens was informed by several aspects of the Perpetrator Mapping Tool developed by the Safe and Together Institute (Mandel, 2015): prioritizing the child through a victim-centered approach in the exploration of the operation of power; focusing on the pattern of coercive actions the person responsible for the exploitation uses; and exploring how the child is impacted by the actions of the person responsible. The outcome of the data analyses was a “synthetic construct”—five forms—that captured the way coerced “self-produced” CSEM is understood in the evidence base.

## Findings

This review identified five forms of children coerced to “self-produce” CSEM: Solicitation; Peer Sexting; Viral Challenge; Sextortion; and Financial Coercion (see Supplemental Appendix A which highlights these critical findings). Table 4 outlines which papers informed each form. The data gathered from the 38 papers reveal the literature’s ambiguity, and at times apparent lack of interest in understanding the pattern of coercive actions children are subjected to. There is clear acknowledgment of those responsible for coercion in particular forms which is absent in other forms, and this impacts the way accountability is constructed across the forms. Further, there is substantial variation among the five forms in relation to either victim-blaming or victim-centered constructions of children coerced to “self-produce” CSEM.

**Table 4.** Summary of Papers Informing Each Form.

CSEM form	Papers that have contributed evidence to each form
The Solicitation form	Caffo et al. (2021); Cooper et al. (2016); Mitchell et al. (2007); Finkelhor et al. (2022); Judge and Saleh (2013); Prat and Jonas (2013); Ringrose et al. (2022); Seto (2013); Sheehan (2016); Smallbone and Wortley (2017); Thorburn et al. (2021); United States Department of Justice (2016); Whittle and Hamilton-Giachritsis (2017); Wolak et al. (2012)
The Peer Sexting form	Cooper et al. (2016); Barr (2016); Gámez-Guadix et al. (2022); Giroux (2021); Graw Leary (2008); Hasinoff (2010); Judge and Saleh (2013); Karaian (2012); Lievens (2012); Macilotti (2020); Meehan (2022); Moritz and Christensen (2020); Patel and Roesch (2022); Primack (2018); Ringrose et al. (2021); Ringrose et al. (2022); Seto (2013); Slane (2013); Strasburger et al. (2019); Thomas (2018); Thorburn et al. (2021); Villacampa (2017); Wolak et al. (2012)
The Viral Challenge form	Grannò et al. (2020); Salter and Hanson (2021); Quayle (2021)
The Sextortion form	Hasinoff (2010); Levine (2022); Macilotti (2020); O’Malley and Holt (2022); Sheehan (2016); United States Department of Justice (2016); Whittle and Hamilton-Giachritsis (2017)
The Financial Coercion form	Açar (2017); Giroux (2021); Hasinoff (2010); Jonsson et al. (2015)

*Note*. CSEM = child sexual exploitation material.

### The Solicitation Form

Fourteen papers contributed to the Solicitation form, which involves adults asking children for sexual pictures, videos or to livestream sexual acts. Only instances that involved an adult directly asking a child for sexual images or videos, without the use of blackmail or financial coercion, are included in the Solicitation form.

#### Solicitation: Methods of Coercion

In this form, perpetrators use a variety of strategies when soliciting coerced “self-produced” CSEM from victims. It is often assumed that perpetrators use technology to trick children by impersonating children (United States Department of Justice, 2016). While this does occur (Sheehan, 2016), Mitchell et al.’s study (2007) indicates that only 5% of solicitation cases involve adults pretending to be children. This is supported by Seto (2013) who argued that solicitation perpetrators do not always impersonate children.

Adult perpetrators may openly send requests for CSEM to a child and these attempts to solicit CSEM can occur at any time during grooming conversations. Ringrose et al. (2022) conducted 37 focus groups with children; one participant explained: “Random older men would send an image and then ask for one in return” (p. 4). This example aligns with Caffo et al.’s (2021) finding that not all perpetrators engage in a friendship-forming stage before sexualizing the conversation, with some introducing their sexual needs early in conversations when soliciting CSEM. Some solicitation perpetrators use persistent demands, insistent questioning, and false promises of privacy to convince children to provide CSEM (Sheehan, 2016).

#### Solicitation: Constructions of Accountability

Applying the Accountability Lens to the Solicitation form reveals that papers published in earlier decades tend to construct children solicited and coerced to “self-produce” CSEM as culpable for their own exploitation. In their systematic review, Prat and Jonas (2013) argued that there is limited information about solicitation due to children being “ashamed” and not telling the truth (p. 10). However, they did not provide data to support this claim. Three years later, Cooper et al. (2016) reported that children increase risk to themselves by joining chat sites, discussing sex online and communicating with people they do not know. These early contributions to the Solicitation form are not victim-centered as they imply that victims are dishonest and responsible for their own abuse.

Subsequent to these early constructions of solicited “self-produced” CSEM, a more victim-centered interpretation began to emerge in the literature. Later studies paid more attention to understanding the coercive and manipulative nature of perpetrators responsible for solicitation (Caffo et al., 2021; Sheehan, 2016; United States Department of Justice, 2016). Furthermore, Ringrose et al.’s (2022) study can be read as refuting the view that children are dishonest, and as demonstrating they truthfully share information about their lived experience of solicitation. These more recently published papers show that over a period of a decade, there has been a significant shift from victim-blaming to victim-centered constructions of coerced “self-produced” CSEM.

### The Peer Sexting Form

Twenty-three papers contributed to the Peer Sexting form. “Sexting” involves using technology to send and receive sexual photos and videos, sometimes referred to as “nudes.” In New Zealand, Thorburn et al. (2021) undertook focus groups with 28 children who said nudes include photos of cleavage, breasts, buttocks, underwear and partially or fully naked bodies. The defining feature is the intention for the images to be sexual. Photos revealing the same amount of skin but non-sexual, such as wearing swimmers at the beach, were not considered nudes by this cohort. A clear distinction emerged in the literature between consensual sexting and coercive sexting, and there are differences in how accountability is constructed in papers contributing to the form.

#### Peer Sexting: Methods of Coercion

This review found that the Peer Sexting literature can be understood in relation to a continuum from consensual sexting to coerced sexting. Some literature constructs consensual sexting between children as an everyday modern form of romantic communication and flirtation (Cooper et al., 2016; Macilotti, 2020; Strasburger et al., 2019; Villacampa, 2017); a way to impress people (Thorburn et al., 2021); a way for children to express themselves (Cooper et al., 2016); and, less frequently, a non-sexual activity done out of curiosity (Wolak et al., 2012). Villacampa’s (2017) survey involving 489 teenagers in Spain found that children often trivialize sexting, a perspective which suggests it has become part of everyday communication for children. Consensual sexting has also been constructed in the literature as being a “safe” alternative to sexual intercourse as it carries no risk of pregnancy or sexually transmitted diseases (Moritz & Christensen, 2020). It has also been claimed that children can experience positive emotions from sexting (Cooper et al., 2016; Primack, 2018; Villacampa, 2017). However, the idea that any Peer Sexting can be truly consensual is also debated in the literature. Thorburn et al. (2021) highlighted the perspective of a child who said that sexting always has an aspect of pressure which indicates that purely consensual sexting is not possible.

Several papers identified children feeling obliged, coerced and pressured to send sexual photos and videos to other children resulting in coerced “self-produced” CSEM. Children in Thorburn et al’s. (2021) study described requests for sexts as being pushy, assertive, persistent, difficult to escape and annoying. These children said refusing to sext could result in peers viewing them as a “prude” or “killjoy,” or as “ugly” and “not interesting” (p. 4). Thomas’s (2018) study thematically analyzed 462 stories posted by teenage girls on a website about their lived experiences related to sexting. The participants recalled male peers getting angry, sending a constant barrage of requests for sexual images and videos and then ceasing communication until the female participated in sexting. Further, the girls said their male peers would promise to keep the messages private or delete them straight away, and others said they did not want to disappoint their partners by withholding sexual images. This pressure and desire to please their partners and avoid negative consequences was a common rationale girls articulated for sexting (Ringrose et al., 2022; Thorburn et al., 2021).

#### Peer Sexting: Constructions of Accountability

Applying the Accountability Lens to papers contributing to the Peer Sexting form appeared to reveal a shift from a victim-blaming to victim-centered construction of accountability over time, as in the Solicitation form. In 2008, Graw Leary concluded children who send their “self-produced pornography” to friends must be held accountable for the criminal element of their activity and described the children as “juvenile sex offenders.” Hasinoff (2010) also viewed children who consensually sext as perpetrators of child sexual abuse and exploitation. Debate has arisen regarding the suitability of prosecuting children for sending sexual photos to peers (Barr, 2016). Authors of more recent papers reject the view that children should be legally prosecuted for sexting, and problematize the criminal label associated with these children (Giroux, 2021; Lieven, 2012; Macilotti, 2020; Primack, 2018; Strasburger, et al., 2019).

Papers published in 2016 uphold a victim-centered approach to sexting by constructing children who have their sexual images shared without permission (“secondary sexting”) as victims, and constructing peers who shared the image as responsible for exploitation. The literature has recognized how those who share sexts without permission cause significant harm to the victims (Primack, 2018), including becoming distressed, feeling humiliated, being bullied and ostracized, being targeted for sexual harassment, being punished by their school and parents and losing control over who has access to images of their body (Meehan, 2022; Thomas, 2018). These victims may also experience sadness, anger, and depression, and some have ended their lives (Cooper et al., 2016; Primack, 2018). Ringrose et al. (2022) acknowledged how schools can become unsafe environments for victims of secondary sexting, who are often made to attend alongside those who share the sexts. Cooper et al. (2016) discussed how secondary sexting can act as an extension of sexual harassment that female students already experience. The capacity of current authors in the field to recognize the victimhood and harm that children can experience within the Peer Sexting form reduces the risk of victims being held responsible for their exploitation and enables peers to be held accountable.

### The Viral Challenge Form

Three publications contributed to an understanding of the Viral Challenge form. Key characteristics of this form involve children uploading videos of themselves onto websites such as YouTube to participate in different viral video challenges. Grannò et al. (2020) analyzed 1,398 YouTube comments left on videos containing “child-produced sexualized imagery” and analyzed YouTube keyword searches for this material. These videos often depicted children participating in viral challenges such as executing certain gymnastic positions or yoga moves whereby their genital area is exposed (Salter & Hanson, 2021). These videos are likely experienced as non-sexual by the children and instead, they most likely think they are playing a safe and rewarding game.

#### Viral Challenge Form: Methods of Coercion

Literature contributing to the Viral Challenge form has been published since 2020 and consistently constructs adults using the comment section of children’s YouTube videos to coerce them into “self-producing” CSEM as perpetrators. Grannò et al. (2020) identified that these perpetrators are adults who intentionally seek out these videos by searching for “child-produced sexual imagery” and encourage children to continue producing more videos, from which perpetrators receive sexual gratification. The analysis of YouTube comments found perpetrators will leave comments on the children’s videos which include requests for specific performances and suggestions about what the children should wear. Perpetrators also verbally reward the child for their performance. Salter and Hanson (2021) discussed how perpetrators use the “comment” function on YouTube to explain to other perpetrators the exact timestamp in the video of when the child accidentally exposes body parts and shares links to other sexual videos of children. Grannò et al. (2020) recognized how perpetrators openly enjoy “inter-predator communication” (p. 9) to exchange ideas in video comment sections.

The Viral Challenge form constructs the website YouTube as a co-offender responsible for facilitating acts of coercion and exploitation. Salter and Hanson (2021) found YouTube’s algorithm identifies perpetrator video preferences and generates a list of suggested videos of other children that include sexual imagery. Likewise, Quayle (2021) found that YouTube’s video algorithm encourages people sexually interested in children to watch videos of minors partially clothed for sexual gratification. These videos are monetized by YouTube through banner advertisements and in this way, the platform appears to be profiteering from child sexual exploitation (Salter & Hanson, 2021).

#### The Viral Challenge Form: Constructions of Accountability

Literature contributing to the Viral Challenge form consistently places responsibility for the exploitation onto perpetrators and the website YouTube, holding them to account. Children are portrayed as young and unaware of what is occurring, overall constructing them as innocent and naïve victims. This is the only form whereby children likely do not realize they are creating sexual material, and it is the only form where no authors construct children as responsible in some way.

### The Sextortion Form

Seven papers identified in this review related to adults who seek CSEM from children and then use this CSEM to blackmail and extort more CSEM. This is referred to as the Sextortion form. Levine (2022) examined investigative data from prior prosecutions and case files of child sexual exploitation in the United States, which illustrated how this form is associated with the most sadistic sexual exploitation experienced by children resulting in significant harm.

#### Sextortion: Method of Coercion

The literature contributing to the Sextortion form indicates that perpetrators use technology to interact with each other and form communities to collaboratively sextort children (United States Department of Justice, 2016). Levine (2022) identified how perpetrators find each other using social media sites and communicate using chat applications such as Discord and Skype to create detailed spreadsheets that delegate offending tasks to different group members. Hierarchies of control exist within these communities, and in some there are formalized processes perpetrators use to request other members to target and sextort a specific child. The examination of case files found that group members within these networks share “how to guides” about sexually exploiting children and avoiding detection by law enforcement. This was also reported in the United States Department of Justice’s (2016) National Threat Assessment, indicating an organized crime approach to exploit children.

Other organized crime methods were identified in the literature. Levine (2022) provided case examples of perpetrator groups that design technology to assist their sextortion, including software to sort profiles of potential victims who are less likely to be cautious. While Hasinoff (2010) reviewed several studies and argued that worrying about perpetrators targeting children whose images they found on the internet was spurious, Levine (2022) found that perpetrators do seek out children whose images they locate on the internet, including children aged between 7 and 14 years who engage in self-harm or display suicidal ideation as they are believed to be more vulnerable to grooming. Likewise, O’Malley and Holt’s (2022) analysis of 152 sextortion perpetrator profiles found that some target children who appear easy to access and manipulate.

Levine (2022) found that after a child is selected, perpetrators follow sequential steps to enact the sextortion, including grooming in an unmoderated internet location; manipulating the victim to “self-produced” CSEM; and then blackmailing the victim to provide more CSEM. Perpetrators sometimes initiate conversations with victims on social media and move to unmoderated internet locations to avoid detection. Then perpetrators manipulate the child into sharing sexual photos, videos or live streaming. Some perpetrators impersonate children to develop a sense of trust with the victim (Levine, 2022; Sheehan, 2016). Perpetrators also lie to children and say they can block the livestreaming from others, and lavish children with compliments when they comply with their demands, ignoring those who do not (Levine, 2022). Sometimes perpetrators use hacking or theft to gather sexual images of children without their knowledge (O’Malley & Holt, 2022).

Levine (2022) also found that perpetrators threaten to send CSEM to the child’s parents, spread the CSEM on the internet or kidnap the child and murder them if they do not continue creating CSEM. In addition to these threats, perpetrators often commit significant acts of abuse such as coercing children to film themselves engaging in sadistic acts. Examples include victimizing a child every day for 4 years, recording children displaying sexually harmful behavior toward their younger siblings and family pets, and recording them self-harming (Levine, 2022; O’Malley & Holt, 2022). The sadistic and coercive nature of perpetrator actions are clearly articulated in the literature contributing to the Sextortion form.

#### The Sextortion Form: Constructions of Accountability

Most of the authors contributing literature to the Sextortion form hold adult perpetrators responsible for the significant harm caused to children. Levine’s (2022) report found that victims of sextortion experienced depression, post-traumatic stress disorder, anxiety, thoughts and plans of suicide, multiple suicide attempts, and dying by suicide. While most papers constructed the children in the Sextortion form as victims, one paper referred to a child who was forced by adult perpetrators to “abuse others” as a “perpetrator” who engaged in “deviant behaviour” such as “bestiality” (O’Malley & Holt, 2022, p. 269). However, the majority of the papers are victim-centered in that they construct children as victims (Levine, 2022; O’Malley & Holt, 2022; Sheehan, 2016; Whittle & Hamilton-Giachritsis, 2017).

### The Financial Coercion Form

Four papers identified perpetrators promising children something of monetary value in exchange for “self-produced” CSEM. In this review, this form of exploitation is labeled the Financial Coercion form. The literature contributing to this form is scarce. There is minimal explanation of the perpetrator’s coercive methods, and all papers position the children as partially responsible for their own abuse without exploring harmful impacts.

#### Financial Coercion: Methods of Coercion

The literature contributing to the Financial Coercion form tends to focus on the victim rather than on the coercive actions of perpetrators using technology to provide children gifts and money to “self-produce” CSEM. Hasinoff (2010) discussed how children “sell” sexual material of themselves on websites. The author acknowledged that this could be the result of coercion or motivated by seeking profit, but then proceeded to focus on the case of a teenage boy who made a “for-profit website” to “perform sex acts” over a webcam for adult men “to profiteer” rather than a case study about coercion. The Turkish National Police (Açar, 2017) constructed this issue as “webcam child prostitution” whereby the “victim simply sells his/her lives sexual images” (p. 98); the authors argued they prefer this construction due to the commercial element involving the child who “exposes himself/herself in a lascivious manner in return for a payment from the offender/consumer” (p. 99). Likewise, Giroux (2021) described children receiving money for their “self-produced” sexual material which they “sell” on the internet, including the case of a 16-year-old receiving $15,000 to $20,000 a month via the website OnlyFans for posting CSEM. Despite recognizing the provision of money in exchange for CSEM, the author did not identify this as a coercive action by perpetrators.

The provision of money and gifts to children in exchange for CSEM as an act of financial coercion by perpetrators is underexplored in the literature. Further, the perspectives of financially coerced children have not been explored making it difficult to identify how they understand this experience. The literature does not focus on the perpetrators’ coercive actions and consequently, the victim children are constructed as responsible for their exploitation.

#### The Financial Coercion Form: Constructions of Accountability

Applying the Accountability Lens to the papers contributing to the Financial Coercion form highlights how perpetrators’ coercive actions are minimized and hidden behind the construction of children as using their agency to undertake “sex work.” Açar (2017), Giroux (2021), and Hasinoff (2010) described children exploited through the Financial Coercion form as “Camboys,” “Camgirls,” or “child prostitutes.” Authors (Giroux, 2021; Hasinoff, 2010) refer to CSEM as “child pornography” that is sold by the victims “for profit.” This aligns with Jonsson et al.’s (2015) study involving 3,432 Swedish high school students who completed a questionnaire which indicated some students “sold sex online” (p. 3). The construction of children as creators and “sellers” of “pornographic” material implies they are in paid employment akin to adults in the sex work industry. This prevents adult perpetrators from being held accountable for financial coercion and exploitation of children. The papers contributing to the Financial Coercion form were published between 2010 to 2021 and during this time there is no apparent progression from victim-blaming to victim-centered constructions of this exploitation.

### The Use of Social Media, Where Coercion Is Not Visible

Several papers in this review relate to children who share sexual photos of themselves on their social media websites and applications, and no coercion is evident. This behavior is characterized by having no clear adult perpetrator and no obvious coercion against the child, therefore it has not been identified as involving a separate form of coercion. Cooper et al. (2016) found children are motivated to share sexual photos of themselves on social media to gain attention, positive reactions from peers, and construct “desirable” visual representations of themselves to use for self-promotion and popularity within their peer group. Karaian (2012) argued this emerging digital social landscape allows children to use technology to publicly share sexual images of themselves on social media while maintaining a sense of privacy.

Despite the behavior being constructed as consensual and initiated by the child, it is not free from negative consequences and potentially still not fully understood. Meehan (2022) heard from one child who explained people leave public comments on peers’ Facebook photos such as: “You’re a whore, you’re just attention seeking” (p. 237). Female children who express their sexuality have been stigmatized and labeled a “lolita, prostitots, kinderwhore, seductive and socially disruptive” (Hasinoff, 2010, p. 223). There is a lack of research available which focuses on children initiating the “self-production” of CSEM on social media with no coercion present and consequently, the available literature provides a fragmented overview of the lived experience of these children. Further research is required to determine whether there is a genuine lack of coercion in this area, or if existing studies have simply not yet identified its presence.

### Children’s Gender and Sexual Orientation

The papers that contributed to this review often explored how victims with binary genders experience different forms of coerced “self-produced” CSEM (Caffo et al., 2021; Giroux, 2021; Grannò et al., 2020; Graw Leary, 2008; Hasinoff, 2010; Jonsson et al., 2015; Mitchell et al., 2007). Participation of children who were not cis-gendered was recorded in few papers, and the impact of their gender concerning the exploitation was not extensively examined (Finkelhor et al., 2022; Meehan, 2022; Ringrose et al., 2022; Thorburn et al., 2021). Female children were often observed to have their sexuality regulated and their exploitation shamed, more than their male peers (Hasinoff, 2010; Karaian, 2012; Meehan, 2022; Thomas, 2018; Thorburn et al., 2021). Overall, females are more vulnerable than their male peers to suffer from coerced “self-produced” CSEM and to also be stigmatized as a result (Giroux, 2021; Grannò et al., 2020; Judge & Saleh, 2013; Meehan, 2022, Ringrose et al., 2022).

Few papers discussed children who were not heterosexual, however, the intersection of these victims’ sexual orientations was not critically explored (Cooper et al., 2016; Ringrose et al., 2022). Papers that did consider diverse genders and sexual orientations often summarized both gender and sexuality as “sexual minorities” and the distinction between how gender diversity and sexuality diversity were experienced requires deeper inquiry (Caffo et al., 2021; Meehan, 2022). How gender identity and sexual orientation of children within all forms of coerced “self-produced” exploitation remains underexplored.

## Limitations

A limitation of this review relates to excluding news media from the selection criteria. Emerging forms of technology-facilitated child sexual abuse and exploitation are often first reported by journalists and then later by academics in peer-reviewed publications. For example, this review indicates it is much easier to learn about perpetrators financially coercing children on the application “OnlyFans” through news media (**Gallier**, 2020) rather than academic journals. The exclusion of news media has resulted in YouTube being identified in this review as profiting from the Viral Challenge form, without acknowledging the multitude of other technology platforms that also profit from the forms of coerced “self-produced” CSEM (National Center on Sexual Exploitation, 2024), which were not identified in the 38 papers included in the review. The decision to exclude news articles due to their lack of scholarly rigor may have limited our presentation of the phenomena. The inclusion period relating to year of publication may also have limited findings of our review as relevant papers may have been published before January 2005.

## Discussion

The identification of five forms of coerced “self-produced” CSEM contributes to a comprehensive and nuanced understanding of this phenomenon. This review has discovered certain coercion strategies influence how children can become excluded from victimhood, and how this has been reconstructed over time. The implications of these findings are explored by identifying ways these five forms of coerced “self-produced” CSEM can inform practice intervention, influence legal frameworks and guide policy development to combat coerced “self-produce” CSEM (summarized in Table 5).

**Table 5.** Implications for Practice, Policy, Legislation, and Research.

**Implications for practice** • A prerequisite to creating safety for children suffering from coerced “self-produced” CSEM, is to first include them in victimhood. • To combat coerced “self-produced” CSEM, child safeguarding organizations will need to become highly capable of constantly tailoring unique responses to each form of coercion identified • Forms of coercion can be explained in training for mandatory reporters of child abuse. **Implications for policy and legislation** • Children would benefit from legal frameworks that disenable a child from being the offender of an offense when they are also the victim. • To reduce financial incentives for technology companies neglecting safeguarding measures, technology companies who financially profit from coerced “self-produced” CSEM needs to be viewed as co-offenders of the exploitation. • Children require enhanced regulation of apps and their content rating. Regular reassessment that considers the user actions and contributions, as well as the frequency of reported child abuse on the app, is required. • Policies need to refrain from terminology that misplaces victims of financially coerced “self-produced” CSEM in the sex work industry. **Implications for future research** • Children within the Financial Coercion form urgently require victim-centered research approaches to understand the coercion by perpetrators, ways safe people can identify Financial Coercion is occurring, effective disruption strategies and the consequential impacts of harm to victims. • Further research is warranted to understand the unique needs and challenges in supporting children of all genders and sexual orientations who are coerced in any form to “self-produce” CSEM. • Further research is needed to determine whether children are independently producing CSEM on social media, or if there is an undiscovered form of coercion that needs responding to.

*Note*. CSEM = child sexual exploitation material.

### Constructions of Victims

The findings of this review demonstrate how certain coercion strategies impact how children are viewed in relation to constructions of victimhood. For example, the use of blackmail correlated with children being constructed as victims of CSE (Levine, 2022; United States Department of Justice, 2016). However, coercion that involved the provision of finances led to children being constructed as “camboys, camboys and prostitutes” (Giroux, 2021; Hasinoff, 2010). It appears that when coercion involves the provision of something of value to the child there can be an assumption that the money or resources “offset” the harm occurring to the child. Regardless of the coercion strategy or perceived agency of the victim, it is the authors’ view that all forms of coerced “self-produced” CSEM constitute child sexual exploitation and cause children significant harm.

### Re-Constructing “Self-Produced” CSEM Over Time

Through applying an Accountability Lens, it was found that constructions of children coerced to “self-produce” CSEM change over time. The literature contributing to each form demonstrated that children are often vilified, or accorded undue agency when a new form of “self-produced” CSEM emerges. For example, authors exploring the Solicitation form once had authors argue children do not tell the truth about being solicited (Prat and Jonas, 2013), while the Peer Sexting form initially promoted the criminalization of children for sexting and viewed them as child sex offenders (Graw Leary, 2008; Hasinoff, 2010). Further, authors contributing to the Sextortion form initially constructed children blackmailed as “perpetrators” (O’Malley & Holt, 2022, p. 269). Over time, the literature contributing to these forms appears to slowly re-construct children as victims who are perpetrated against by adult offenders. This process of reconstruction seems to occur across decades and has not begun at all in the literature contributing to the Financial Coercion form.

### Implications for Practice

A prerequisite for creating safety for children suffering from coerced “self-produced” CSEM, is to first include them in the category of “victim”. In response to children wrongfully constructed as responsible for becoming coerced and exploited, child safeguarding organizations would benefit from assessment processes capable of prompting practitioners to reconsider any problematic assumptions about children’s agency regarding coerced “self-produced” CSEM. This process and outcome are necessary when building workforce capacity to focus on changing the behavior of those responsible for the coercion.

To combat coerced “self-produced” CSEM, child safeguarding organizations will need to become highly capable of constantly tailoring unique responses to each form of coercion identified. Each form of coercion entails manipulative processes that can be anticipated and prevented through a multiagency approach to disrupt each coercive action. Prevention and disruption responses will require regular adaption to combat expected technological advancements. This demands a continuous and vigilant effort to identify solutions to keep children safe from each form of coercion.

The authors propose that children can be better supported when the services that assist them, understand the exploitation they might be at risk of. Relevant information about each form of coercion can be integrated into training material for mandatory reporters of known or suspected child abuse. It is anticipated this will enhance vigilance and enable adults to create informed responses to coerced “self-produced” CSEM.

### Implications for Policy and Legislation

The findings of this review support policy and legal frameworks capable of creating perpetrator accountability and promoting a regulated technology industry that prioritizes the safety of children.

There remain existing legislative frameworks that allow for a child to be charged and convicted of creating CSEM when the material is of themselves (O’Connor et al., 2017). Perpetrators can leverage these existing legislative frameworks to divert criminal responsibility onto the child and silence disclosures by making children fear prosecution. To remove this barrier to children accessing safety, the authors support legislative amendments that explicitly state a child cannot be classified as an offender when they are already the victim. This could increase children’s willingness to seek support from law enforcement and mandatory reporters after being coerced to “self-produce” CSEM, and help law enforcement reserve prosecuting resources for the person responsible for using coercion.

In every form of coerced “self-produced” CSEM, technology facilitates the exploitation and, at times, technology platforms can be thought about as co-offenders. The authors argue it would be in children’s best interests for legal frameworks to hold technology companies responsible when they financially benefit from coerced “self-produced” CSEM. For example, it is known that children are exploited on OnlyFans (Gallier, 2020; Giroux, 2021) as part of the Financial Coercion form, and OnlyFans collects a 20% commission each time this occurs (OnlyFans, 2024). Children need legislation that removes technology companies’ financial incentives for insufficient child safeguarding practices.

This review calls for software applications (apps) to have enhanced regulation. Currently, an app will display the minimum maturity level or age, based on the app developer’s content and will align with the content rating standards in the region the app is being accessed (Google, 2024). These ratings are determined by the app’s design, instead of what app users contribute to the app (Levine, 2022); this provides misleading guidance for caregivers when deciding what apps their children are safe to use. For example, the Roblox website states their app rating is for children 12 years and older (Roblox, 2024), despite the National Centre on Sexual Exploitation (2024) confirming “countless children have been sexually abused and exploited by predators they met on Roblox.” Policy reform is required to enforce app’s content ratings to be regularly reviewed and made to reflect the contributions and actions of those using the apps, as well as the frequency of reported child abuse on the app. This would more accurately inform caregivers and children about what apps pose a significant risk of exploitation to children.

This review has demonstrated how terminology can influence the way in which a problem is understood, highlighting the need for policies safeguarding children from the Financial Coercion form to remove lexicon that constructs children as part of the sex work industry. Future policy reform needs to consistently construct these children as victims, to ensure their vulnerability and need for protection is understood.

### Further Research

Further research is required to explore methods used by adult perpetrators against children in the Financial Coercion form. Applying the Accountability Lens, it is clear that authors researching this form continue to view child sexual exploitation as employment, incorrectly implying that it is consensual and that victims have agency and control. With the increasing volume of children becoming active on sites such as OnlyFans, it is imperative that further research acknowledges the coercive experience of the children and recasts them as victims. In addition, the lived experience of children within the Financial Coercion form urgently requires more research informed by a victim-centered approach to understand the methods of coercion used by perpetrators and the consequential impacts of harm on victims. Further research is warranted to understand the unique needs and challenges in supporting children of all genders and sexual orientations who are coerced in any form to “self-produce” CSEM. The five forms of coercion identified can all be better understood in future research by collecting information from children specifically on each form of coercion. As new forms of coerced “self-produced” CSEM emerge it is important for researchers to adopt a victim-centered lens to harness opportunities from the outset to reduce risk and harm.

## Conclusion

This review has found that “coerced ‘self-produced’ CSEM” is an umbrella term that encapsulates a diversity of children’s experiences of being manipulated to create CSEM. The 38 papers reviewed contributed to five forms. Applying the Accountability Lens, it was revealed that when new forms of coercion emerge, children are at risk of being constructed as responsible for exploitation. We assert that children are never responsible for their own exploitation and that further victim-centered research focused on the Financial Coercion form is urgently required.

## Supplemental Material

sj-docx-1-tva-10.1177_15248380241271376 – Supplemental material for Five Forms of Coerced “Self-Produced” Child Sexual Exploitation Material: A Critical Interpretive Synthesis Supplemental material, sj-docx-1-tva-10.1177_15248380241271376 for Five Forms of Coerced “Self-Produced” Child Sexual Exploitation Material: A Critical Interpretive Synthesis by Genevieve Bloxsom, Gemma McKibbin, Cathy Humphreys, Jennifer Davidson and Nick Halfpenny in Trauma, Violence, & Abuse
