# Supplementary material for: Five Forms of Coerced “Self-Produced” Child Sexual Exploitation Material: A Critical Interpretive Synthesis
Source: Trauma Violence Abuse. 2024 Sep 8;25(5):4230–44. doi: 10.1177/15248380241271376 (PMC11545210; doi:10.1177/15248380241271376)

| Implications for practice   - A prerequisite to creating safety for children suffering from coerced “self-produced” CSEM, is to first include them in victimhood. - To combat coerced “self-produced” CSEM, child safeguarding organisations will need to become highly capable of constantly tailoring unique responses to each form of coercion identified - Forms of coercion can be explained in training for mandatory reporters of child abuse.   Implications for policy and legislation   - Children would benefit from legal frameworks that disenable a child from being the offender of an offence, when they are also the victim. - To reduce financial incentives for technology companies neglecting safeguarding measures, technology companies who financially profit from coerced “self-produced” CSEM need to be viewed as co-offenders of the exploitation. - Children require enhanced regulation of apps and their content rating. Regular reassessment that considers the user actions and contributions, as well as the frequency of reported child abuse on the app is required. - Policies need to refrain from terminology that misplaces victims of financially coerced “self-produced” CSEM in the sex work industry.   Implications for future research   - Children within the Financial Coercion form urgently require victim-centred research approaches to understand the coercion by perpetrators, ways safe people can identify financial coercion is occurring, effective disruption strategies and the consequential impacts of harm to victims. - Further research is warranted to understand the unique needs and challenges in supporting children of all genders and sexual orientations who are coerced in any form to “self-produce” CSEM. - Further research is needed to determine whether children are independently producing CSEM on social media, or if there is an undiscovered form of coercion that needs responding to. |
| --- |


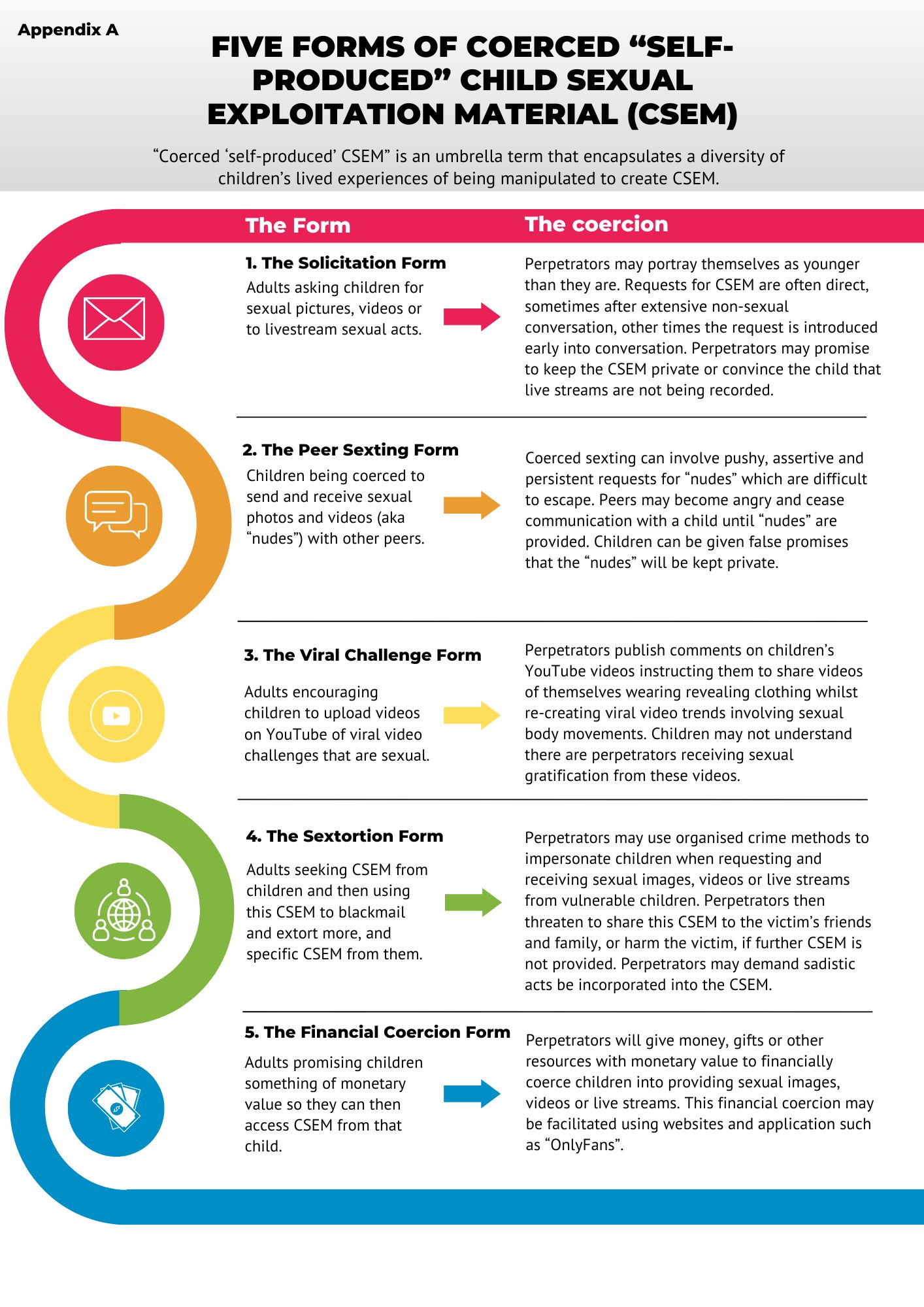

Supplement: sj-docx-1-tva-10.1177_15248380241271376 – Supplemental material for Five Forms of Coerced “Self-Produced” Child Sexual Exploitation Material: A Critical Interpretive Synthesis [file sj-docx-1-tva-10.1177_15248380241271376.docx]
